# Weight Status and Associated Risk Factors of Mealtime Behaviours among Children with Autism Spectrum Disorder

**DOI:** 10.3390/children9070927

**Published:** 2022-06-21

**Authors:** Maizatul Naqiah Zulkifli, Masne Kadar, Nur Hana Hamzaid

**Affiliations:** 1 Dietetics Programme, Centre for Rehabilitation and Special Needs (iCaRehab), Faculty of Health Sciences, Universiti Kebangsaan Malaysia, Jalan Raja Muda Abdul Aziz, Kuala Lumpur 50300, Malaysia; m.naqiah@gmail.com; 2 Department of Dietetics and Food Services, Sungai Buloh Hospital, Ministry of Health, Jalan Hospital, Sungai Buloh 47000, Malaysia; 3 Occupational Therapy Programme, Centre for Rehabilitation and Special Needs (iCaRehab), Faculty of Health Sciences, Universiti Kebangsaan Malaysia, Jalan Raja Muda Abdul Aziz, Kuala Lumpur 50300, Malaysia; masne_kadar@ukm.edu.my; 4 Department of Dietetics and Food Services, UKM Specialist Children’s Hospital, Universiti Kebangsaan Malaysia, Jalan Yaacob Latif, Cheras, Kuala Lumpur 56000, Malaysia

**Keywords:** children, autism spectrum disorder, mealtime behaviours, weight status, oral sensory processing

## Abstract

Atypical mealtime behaviours in children with autism spectrum disorder (ASD) have been linked with oral sensory hypersensitivity that may contribute to food selectivity and weight issues. This cross-sectional study aims to determine the association between atypical mealtime behaviours and associated risk factors besides exploring the prevalence of overweight and obesity among Malaysian children with ASD in community settings. A total of 150 children with ASD aged 3–11 years participated in this study. A self-administered questionnaire on sociodemographic variables, mealtime behaviours and oral sensory processing was completed by the caregivers. The prevalence of overweight in the study samples was 18.5%, whereas obesity was 20.0%. In a multivariate analysis model, oral sensory processing (B = 0.608, 95% CI = 0.493, 0.722, *p* < 0.001), severity of autism symptoms (moderate and severe) (B = 2.585, 95% CI = 0.722, 4.448, *p* = 0.007) and younger children with ASD (B = −0.694, 95% CI = −1.189, −0.199, *p* = 0.006) were found as independent predictors of atypical mealtime behaviours. Children with ASD aged 3–11 years old have a higher prevalence of being overweight and obese, thus requiring regular anthropometric screening in community settings by relevant healthcare professionals. Furthermore, a relationship was found between oral sensory difficulties and atypical mealtime behaviours in children with ASD. A multidisciplinary approach is crucial in the overall management of food selectivity in this population.

## 1. Introduction

Autism spectrum disorder (ASD) refers to a group of neurodevelopmental disorders that include social communication problems as well as repetitive and rigid behaviours [1]. In the United States (US), the prevalence rate is 1 in 59 children with ASD [2]. To date, the prevalence of ASD in Malaysia has yet to be determined. However, a feasibility study conducted under the Ministry of Health Malaysia (MOH) among Malaysian children with ASD aged 18–36 months using the Modified Checklist for Autism in Toddlers (M-CHAT) as an instrument for screening at various health clinics found that the prevalence of children with ASD is estimated at 1.6 in 1000 children [3].

Atypical mealtime behaviours are commonly reported among children with ASD associated with food fussiness or highly selective nature in food intake as well as refusal to try new foods, which was estimated to range from 46% to 89% [4]. Recent findings from a cross-sectional study undertaken at a tertiary healthcare centre located in Kuala Lumpur found that 91.4% of children with ASD aged 2–18 years had nutrition-related behavioural problems associated with food selectivity, leading to a negative impact on their body mass index (BMI) status [5].

At present, the meaning of food selectivity varies due to different terms and measurement tools used for evaluation besides longitudinal studies needed to determine the age range on the occurrence of eating problems in children [6]. Clinically, Bryant-Waugh et al. [7] classified children’s eating disorder into three aspects, namely (i) limited dietary ranges that focus on specific food brands; (ii) avoiding trying new foods associated with neophobia symptoms; and (iii) rejecting a particular type of food based on texture, taste or smell. Cermak, Curtin and Bandini [8] reported that selective eating in children with ASD is associated with reluctance to eat unfamiliar food, limited dietary ranges and obsession with certain foods, as well as high selectiveness on food groups, such as preferences for high carbohydrate sources over other food groups.

Increasing evidence suggests that children with ASD are more likely to develop and demonstrate feeding issues than children with typical development (TD), such as refusing to try new foods [9,10] and being highly selective about texture, temperature, brand, or food recipe [11,12,13], as well as a limited quantity of food choices [10,14,15] that may create a negative atmosphere and stress among caregivers during mealtimes. According to a review study, the ratio of children with ASD who have eating-related problems and risk of nutrient inadequacy is five times more compared to children with TD [16]. Hence, targeting the underlying issue linked to food selectivity is crucial to minimise long-term health consequences in this population.

Moreover, selective eaters among children with ASD have tendencies to develop oral sensory sensitivities to food textures, tastes or smells causing them to consume only particular food at each time [17,18,19]. A study by Leekam et al. [20] showed that 90% of children with ASD have a different oral and visual sensory processing compared to children with other disabilities. This is further reinforced by the latest diagnostic criteria based on the 5th Edition Diagnosis and Statistical Manual of Mental Disorders (DSM-5), elucidating hypo- or hypersensitivity manifests as one of the features of ASD [1]. A review by Boudjarane et al. [21] also demonstrated that there was evidence of sensory difficulties related to smell and taste among individuals with ASD. Furthermore, taste and smell stimuli are often neglected in the research activity related to sensory processing, although these two sensory aspects have important effects on the daily life of individuals including eating and social-related behaviours. In addition, a recent study by Riccio et al. [22] reported that children with ASD with food selectivity aged between 2 and 11 years exhibited a bitter sensitivity associated with variation of gene TAS2R38, with mainly PROP-taster phenotype that results in aversion and rejection of certain foods, including green leafy vegetables and fruits, compared to non-selective ASD. 

Several risk factors may have contributed to the atypical mealtime behaviours in children with ASD, including oral sensory processing difficulties [19,23,24], parental feeding practices [25], oral-motor problems [12,26] and gastrointestinal (GI) symptoms [27]. Nevertheless, there are still inconsistent findings related to age factors and the severity of ASD symptoms. For instance, from Malaysian perspective, Eow et al. [28] conducted a study at an autism early intervention centre located in Kuala Lumpur and discovered that 78.1% of Malaysian children with ASD aged 3–7 years had severe ASD symptoms (total score ≥ 15) according to the Social Communication Questionnaire (SCQ). Thus, it is crucial to evaluate eating behaviours among Malaysian children with ASD, as prior research has shown that the severity of ASD symptoms may contribute to mealtime difficulties [29], whereas age factors could also exacerbate the atypical mealtime behaviours in children with ASD [30,31]. 

On the other hand, there are discrepancies in the previous findings regarding weight status; for example, children with ASD in the US have a higher prevalence of overweight (26–42.4% vs.22–26%) and obesity (17–30.4% vs. 9–23.6%) compared to children with TD [10,32,33,34]. A few Asian countries also reported an increased proportion of overweight or obesity among children with ASD, including China (31.5%) and Turkey (58.5%) [35,36]. Conversely, a higher percentage of children with ASD has been found to be underweight (20–23.4% vs. 9–12.6%) than TD peers in other countries [37,38].

Therefore, the objectives of the present study are to evaluate the relationship between oral sensory processing and mealtime behaviours and to determine the associated risk factors for mealtime behaviours. Furthermore, this study aims to determine the prevalence of overweight and obesity in children with ASD aged 3–11 years in local community settings. It was hypothesised that there is a significant relationship between oral sensory processing and mealtime behaviours in children with ASD. Additionally, oral sensory processing, age, severity of ASD symptoms and health-related comorbidities (e.g., GI symptoms and attention deficit hyperactive disorder (ADHD)) would predict the episodes of atypical mealtime behaviours among children with ASD. 

## 2. Materials and Methods

### 2.1. Participants

This is a cross-sectional study that involved caregivers and children with ASD aged 3–11 years from 16 community-based rehabilitation (CBR) centres under the Department of Social Welfare, four autism early intervention centres and two autism societies managed by the non-government organisation (NGO) located in the central zone comprising Selangor, Federal Territory of Kuala Lumpur, and Negeri Sembilan. Participants were recruited based on the cluster sampling technique. At the first stage, the target population was divided into smaller groups known as clusters according to several zones (north, south, central, east, Sabah, and Sarawak). The central zone was chosen for this study due to population density and logistic facilities. The second stage involved selecting the districts and certain regions in the central zone. Finally, the CBR and autism early intervention centres in each cluster were randomly selected based on the list of the centres available.

Sample size calculation was done based on the formula for survey study, *n* = [(Z_α/2_)^2^ *p*(1 − *p*)]/*d*^2^], whereby Z is the 95% confidence interval (1.96), *p* is the estimated proportion from the previous literature, 0.22 [5] and *d* is precision ranging between 3 and 10% [39] Hence, the final sample size was 150 with the consideration of an additional 10% calculation for the drop-out rate.

This study had obtained ethical approval from the Research Ethics Committee of the National University of Malaysia (Reference No.: UKM PPI/111/8/JEP-2020-475). Permission was also acquired from the Department of Social Welfare (Reference No.: JKMM 100/12/5/2:2021/002), and NGO centres. Before data collection, relevant CBR and autism early intervention centres were contacted and informed. Informed consent was taken from the caregivers. Self-administered questionnaires were completed by the caregivers.

### 2.2. Measurements

#### 2.2.1. Sociodemographic Data

Sociodemographic information included the personal details of the caregivers and the child. For the child with ASD, information was collected including date of birth, age, gender, ethnicity, category of ASD symptoms, position in the family and medical information. Information of caregivers consists of age, marital status, age of mother at birth of the child with ASD, medical history or illness during pregnancy, education level, occupation and monthly household income.

#### 2.2.2. Anthropometric

Stadiometer (SECA 217) and multi-function platform weighing scale (SECA 674) were used to measure the height and weight of the children with ASD. Meanwhile, the BMI was calculated using measured weight and height and then plotted on the Centre for Disease Control and Prevention (CDC) growth charts to identify percentiles by gender and age. Based on the CDC NCHS growth chart (2000), there are four categories of BMI according to the age–gender of children and adolescents aged from 2 to 20 years that can be classified into underweight (<5th percentiles), normal weight (5th–84th percentiles), overweight weight (85th–94th percentiles) and obesity (≥95th percentiles) to assess children’s weight status [40].

#### 2.2.3. Mealtime Behaviours

The Brief Autism Mealtime Behaviour Inventory (BAMBI) is an 18-item caregiver-reported questionnaire [41] used to evaluate atypical mealtime behaviours in children with ASD aged from 3 to 11 years by assessing three main domains namely food refusal, limited food choices and ASD characteristics. The original BAMBI questionnaire has a good internal consistency, Cronbach’s alpha value of 0.88, and scoring based on a 5-Likert scale; 1 is considered never, while 5 represents almost every meal. A higher value indicates that children with ASD potentially have behavioural disorders while eating. Permission to use BAMBI for this study was obtained from the original author, Dr. Colleen Taylor Lukens, through electronic mail. On the other hand, the BAMBI was translated into Malay language and used among Malaysian children with ASD. The Malay version of BAMBI demonstrated good internal consistency reliability with a Cronbach’s alpha value of 0.83 [5].

#### 2.2.4. Oral Sensory Processing

Child Sensory Profile-2 (CSP-2) is an 86-item parental report [42] that assesses sensory processing difficulties in children aged 3–14 years. CSP-2 contains several domains for assessment consisting of four sensory patterns (seeking, avoiding, sensitivity and registration), six sensory systems (oral, touch, visual, auditory, movement and body position), and sensory-associated behaviours (social–emotional, attentional and conduct). Each domain has an individual total score and an overall total score with a 5-Likert scale; 1 represents never/rarely, and 5 is considered as almost every meal. For this study, only the oral sensory processing (OSP) domain was selected, consisting of 10-item following the study objectives. Permission to use the CSP-2 questionnaire for this research was obtained from the original author, Professor Winnie Dunn, through email. The CSP-2 questionnaire was purchased according to the study sample size from a local authorised distributor company.

The original domain of OSP 10-item was translated into the Malay language by two individuals: one person was an independent professional translator, and another person was a healthcare professional. Both individuals are fluent in English and Malay languages. The OSP Malay version was then back-translated into English by a bilingual professional translator who was unaware of the questionnaire. The next process was content validation of the OSP Malay version by several panel experts consisting of experienced occupational therapists (OTs), followed by a brief cognitive interview with 15 caregivers who have children with a confirmed diagnosis of ASD aged between 3 and 11 years to obtain the understanding and clarity of the OSP in Malay version. In the current study, Cronbach’s alpha for the OSP domain was good (0.85), while the intra-class correlation coefficient (ICC) for test–retest (*n* = 28) was satisfactory by 0.90.

### 2.3. Statistical Analysis

Statistical analyses were carried out using IBM SPSS Statistics 20.0 (IBM Corp., Armonk, NY, USA). Descriptive analyses such as frequency, mean, standard deviation and percentage were used to assess sociodemographic information, weight status, the prevalence of atypical mealtime behaviours and oral sensory processing difficulties. The normality was analysed using the Kolmogorov–Smirnov test, and the skewness value/standard error was in the range of ±2.5. All probability values, *p* < 0.05, were significant with a 95% confidence interval (CI).

The Pearson correlation test was used to evaluate the relationships between the total BAMBI scores and the BAMBI subdomain with the total OSP scores. Furthermore, the multiple linear regression analysis was performed to predict the relationship between the total OSP scores, age, ASD symptoms (moderate and severe), and health-related comorbidities with the total BAMBI scores. The variance inflation factor (VIF) was used to examine multicollinearity, in which the value should be <10 [43]. No issues were found as the VIF values from the current study were within 1.005–1.033.

## 3. Results

The majority of them were males (85.3%), while the remaining were females (14.7%). The mean age of the children with ASD who participated in this study was 7.2 ± 1.9 years. According to parental reports, moderate ASD symptoms occupied the highest percentage of 47.3%, followed by mild ASD symptoms at 43.3%, while 9.3% were in severe symptoms. Notably, the child’s severity of ASD symptoms was undertaken during previous clinical assessment with the child developmental specialist, paediatrician, psychiatrist or child clinical psychologist in the clinic or hospital. About 19.3% of children with ASD had comorbid attention deficit hyperactive disorder (ADHD) symptoms, whereas 12.7% had gastrointestinal symptoms such as constipation, diarrhoea, abdominal pain or vomiting. In terms of medical complications, 16.0% of the mothers had gestational diabetes (GDM) or type 2 diabetes during pregnancy of the child with ASD. More than half of the mothers (54.7%) had education at the tertiary levels. Nevertheless, about 61.3% of the participant families’ monthly household income falls within the lower socioeconomic group, in which their monthly income is equal to or less than MYR 4000 (≈USD 953.20). Table 1 describes the sociodemographic characteristics of the participants.

Complete anthropometric records were available for 135 children, of which 20% were obese with BMI ≥ 95th percentiles, while 18.5% had BMI between 85th and 94th percentiles, which can be considered as overweight. The prevalence of overweight and obesity in children with ASD aged 3–11 years in community settings was 38.5%. Table 2 shows the weight status according to BMI for children with ASD.

A higher number of children with ASD (96.0%) had atypical eating behaviours. More than half of those children had a problem in oral sensory processing (53.3%), in which 34.0% of the sample were in the category of more than others, whereas 19.3% of the children were classified as having much more than others. Table 3 depicts the frequency of atypical mealtime behaviours and oral sensory processing difficulties among children with ASD.

There were positive and moderate relationships with significant values between the total score of oral sensory processing and total score of BAMBI, *r* (150) = 0.65, *p* < 0.001, the sub-domain of BAMBI, which was limited variety of food, *r* (150) = 0.64, *p* < 0.001, as well as food refusal, *r_s_* (150) = 0.45, *p* < 0.001 (Table 4). Notably, the mean BAMBI score for children with ASD was 44.63 (SD = 8.02). 

According to age groups, there was a significant difference in BAMBI score between the younger age (46.12 (SD = 8.00; *n* = 66)) and older age (43.46 (SD = 7.88; *n* = 84)) groups, *p* = 0.044, based on independent samples T-test. However, oral sensory processing scores among the two groups did not differ significantly, *p* = 0.143.

The results of the simple linear regression are shown in Table 5. In the multiple linear regression model for atypical mealtime behaviours, four variables with *p* < 0.05 in the simple linear regression model were examined. Three predictors concerning mealtime behaviours among children with ASD were found statistically significant through the multiple linear regression stepwise method. Children with ASD who had atypical oral sensory processing yielded a higher score of BAMBI (B = 0.608, 95% CI = 0.493, 0.722, *p* < 0.001). Severity of ASD symptoms (moderate and severe) predicted higher BAMBI total score in children with ASD (B = 2.585, 95% CI = 0.722, 4.448, *p* = 0.007). Conversely, children with ASD at younger age had a higher BAMBI score associated with atypical behaviours during mealtime (B = −0.694, 95% CI = −1.189, −0.199, *p* = 0.006). In combination, the three predictors variables explained 49.4% of the variability in the total score of BAMBI (*F* = 47.118, *p* < 0.001; *R^2^* = 0.494, adjusted *R^2^* = 0.483). According to Cohen’s effect size, a combined effect of this magnitude can be considered large, *f^2^* = 0.976 [43]. Table 6 describes the results of the multiple linear regression on the associated risk factors with mealtime behaviours.

## 4. Discussion

This study provides a deeper understanding of the prevalence of overweight and obesity, as well as the atypical mealtime behaviours, and its associated risk factors in Malaysian children with ASD aged 3–11 years old in community settings. Findings from the current study demonstrated that 18.5% and 20.0% of children with ASD were overweight and obese, respectively, thus reflecting the overall overweight and obesity status at 38.5%. These prevalence rates were higher compared to TD children in the general population aged 5–17 years, in which the rate of overweight was 15.0%, while obese was 14.8% based on recent data from the National Health and Morbidity Study (NHMS) [44].

The prevalence of overweight and obesity in the present study was slightly higher compared to that obtained by Xia et al. [35] in China, which reported 31.5% overweight or obese children with ASD aged 2–9 years. Meanwhile, the US also reported a higher prevalence rate of ASD group aged from 4 to 17 years for the overweight category (42.4% vs. 26.1%) and obesity (21.4% vs. 12.0%) compared to children with TD [34]. A recent meta-analysis study by Zheng et al. [45] concluded that the prevalence of obesity is significant for autistic individuals, with an odds ratio of 1.84 higher than that for typical individuals.

One of the risk factors associated with overweight and obesity among children with ASD was extremely picky eating due to atypical sensory profiles, thus leading to dietary and nutritional imbalances [46]. These problems can have a negative impact on the weight status of children with ASD. Furthermore, prior research found that over 90% of children with ASD showed difficulties in oral and visual sensory processing when compared to other children with disabilities [20]. In the present study, more than half of the samples had oral sensory difficulties (53.3%), of which 34.0% were in more than others category, while 19.3% were much more than others. According to Bennetto et al. [47], children with ASD have a poor sense of smell and taste especially for sour and bitter tastes compared to children with TD but can detect sweet and salty tastes well. For instance, a study by Riccio et al. [22] demonstrated that there was an association between food selectivity and bitter taste sensitivity related to TAS2R38 genetics in children with ASD that could affect their food preferences. Selective eating in children with ASD has been linked with a higher intake of energy-dense food including processed foods, sweetened beverages and junk foods, as well as lower fruit and/or vegetable intake in the daily diet, leading to weight gain episodes [10,33].

The present findings revealed that the majority of children with ASD aged 3–11 years (96%) had atypical mealtime behaviours. The increased percentage rate might be due to data collection that was carried out during the coronavirus disease 19 (COVID-19) pandemic, which led the government to enforce the movement control order nationwide and most of the follow-up treatment and therapy of children with ASD at clinic or hospital had to be postponed. Therefore, this possible reason could influence more episodes of challenging behaviours among children with ASD. 

Additionally, the results of this study showed a positive and moderate relationship between the total score of oral sensory processing and challenging mealtime behaviours through the BAMBI tool, *p* < 0.001. Multiple linear regression analysis showed that oral sensory processing score significantly contributed to the substantial variance in atypical mealtime behaviours in children with ASD aged 3–11 years. Wang et al. [48] described that the taste/smell sensitivity domain is the major contributor to atypical mealtime behaviours in children with ASD by 24% compared to other sensory domains in short sensory profile (SSP) assessment. Leader et al. [27] also reported that the SSP score is a contributing risk factor for food selectivity in children with ASD with the majority of the subjects classified as having definite differences (85.3%) in sensory stimuli. This is further highlighted by research that investigated the relationship between oral sensory issues and challenging behaviours during mealtimes associated with limited food repertoire [19,23,49]. In addition, food rejection is significant among children with ASD compared to those with TD and is also influenced by sensory characteristics such as textures, smell/taste, mixed foods, brands and shape [17].

Another significant risk factor in this study was the age factor. Younger children with ASD were observed to exhibit more frequent atypical behaviours during mealtimes. This finding was in line with the longitudinal study by Emond et al. [50], reporting that at 15 months of age, children with ASD had decreased food choices, and upon entering the age of 2 years, eating patterns were different from the rest of their family members. A cross-sectional study of children with ASD with a large sample size (*n* = 1112) revealed that children often display the most challenging mealtime behaviours at the age between 1 and 3 years [29]. Bandini et al. [31] observed the eating patterns of 18 children with ASD at childhood (mean age: 7 years) and during adolescence (mean age: 13 years), which reported a decrease in food refusal episode and a significant improvement in food habits by accepting a variety of food textures and food mixed as age increased. However, restricted food repertoire was still persistent throughout the cohort period. Furthermore, food fussiness commonly occurs in children with TD, and the behaviour was reported to peak between the age of 2 and 6 years [51]. Contrarily, a cross-sectional study involving 141 children with ASD aged 3–9 years found that age did not influence food selectivity [52]. Parental-report outcomes also revealed that sensory difficulties did not vary between the age of 2 and 8 years and remained constant in children with ASD based on the longitudinal study observed by McCormick et al. [53]. These scenarios suggest that the diet of children with ASD is unique and different for each individual. Thus, early detection of nutrition-related problems using validated and reliable instruments is critical for preventing more serious health consequences in the future associated with micronutrient inadequacies [54,55].

Furthermore, the findings of this study demonstrated that ASD symptoms (moderate and severe) have a substantial impact on atypical mealtime behaviours in children with ASD. For instance, Postorino et al. [56] presented that children with ASD who were highly selective (consumed fewer than 10 types of food) possessed more severity in ASD symptoms than the ASD group with typical eating patterns (consumed more than 20 types of food). However, a discrepancy was noted in which the overall score of the Childhood Autism Rating Scale (CARS), a behavioural assessment tool used in determining ASD symptoms, was shown to have no significant correlation with mealtime behaviours in children with ASD aged from 2 to 12 years [57]. The findings from the present study should be interpreted with caution since ASD symptoms were obtained from parental reports. There was no clinical evaluation or use of specific instruments to further validate the severity of ASD symptoms for the participated children.

Several limitations had been identified in this study. First, the causal effects of the relationships between oral sensory processing, the severity of ASD symptoms and age factor with atypical mealtime behaviours could not be confirmed because the current study employed a cross-sectional study design. Thus, cohort studies are needed to corroborate the contributing factors associated with mealtime behaviours in children with ASD. Second, data collection was based on parental reports on a child’s mealtime behaviours and oral sensory difficulties that could potentially influence memory bias. Next, a TD control group was not included in this present study. A comparison group could be useful in future studies to distinguish between children with feeding and sensory difficulties and those without these issues. Furthermore, assessment of dietary intake in children with ASD is crucial for future research as an important variable since the prior research findings have shown that food selectivity could further increase the risk of nutrient inadequacy in children with ASD on a long-term basis and therefore warrants further investigations.

## 5. Conclusions 

In conclusion, this study contributes to the understanding of the relationships between challenging mealtime behaviours and associated risk factors comprising oral sensory difficulties, age and severity of ASD symptoms. Moreover, overweight and obesity are prevalent in Malaysian ASD children aged 3–11 years in community settings. Furthermore, since eating involves multisensory skills, children with ASD tend to be anxious and overwhelmed especially when they have issues with sensory processing difficulties. Therefore, multidisciplinary collaboration needs to be done involving registered dietitians, occupational therapists, speech-language pathologists, clinical psychologists and/or behavioural therapists in the overall management of food selectivity and food avoidance among children with ASD. Additionally, regular anthropometric screening should be conducted by relevant healthcare professionals to evaluate the nutritional status of children with ASD and facilitate proper intervention strategies.

## Figures and Tables

**Table 1:** Sociodemographic characteristics of children with ASD and their caregivers (*n* = 150).

Characteristics	*n* (%)	Mean ± SD
**Children with ASD**		
**Ethnicity**		
Malay	134 (89.3)	
Native people from Sabah/Sarawak	7 (4.7)	
Chinese	5 (3.3)	
Indian	4 (2.7)	
**Age (years)**		7.2 ± 1.9
3–6	66 (44.0)	
7–11	84 (56.0)	
**Gender**		
Male	128 (85.3)	
Female	22 (14.7)	
**ASD symptoms**		
Mild	65 (43.3)	
Moderate	71 (47.3)	
Severe	14 (9.3)	
**Position in the family**		
First child	63 (42.0)	
Second child	38 (25.3)	
Third child and above	49 (32.7)	
**Health-related comorbidities**		
ADHD	29 (19.3)	
Epilepsy	3 (2.0)	
Gastrointestinal symptoms (e.g., constipation, diarrhoea, abdominal pain, vomiting)	19 (12.7)	
2 or more health-related co-morbidities	17 (11.3)	
**Caregivers**		
Age (years)		38.0 ± 7.0 ^a^
**Mother’s pregnancy complication**		
Without GDM/DM Type 2	126 (84.0)	
GDM/DM Type 2	24 (16.0)	
**Mother’s age during child given birth (ASD): years**		
≤20	6 (4.1)	
21–34	115 (77.7)	
35–39	21 (14.2)	
≥40	5 (3.4)	
**Mother’s educational level**		
Primary education	4 (2.7)	
Secondary education	48 (32.0)	
Post-secondary education	15 (10.0)	
Tertiary education	82 (54.7)	
**Father’s educational level**		
Primary education	3 (2.0)	
Secondary education	54 (36.0)	
Post-secondary education	31 (20.7)	
Tertiary education	58 (38.7)	
Monthly household income (* MYR)		
≤4000 (low)	92 (61.3)	
4001–8000 (moderate)	42 (28.0)	
8001–12,000 (moderate–high)	8 (5.3)	
>12,000 (high)	7 (4.7)	
**CBR/ NGO autism intervention centres**		
CBR Centres in Selangor	53 (35.3)	
CBR Centres in Kuala Lumpur	20 (13.3)	
CBR Centres in Negeri Sembilan	14 (9.3)	
NGO autism centres in Kuala Lumpur and Selangor	63 (42.0)	

* MYR = Malaysian Ringgit: USD 1 = MYR 4.20 as per 24 February 2022. ^a^ Median ± IQR; ASD—autism spectrum disorder; ADHD—attention deficit hyperactive disorder; GDM—gestational diabetes mellitus; DM—diabetes mellitus; CBR—Community-based rehabilitation; NGO- non-governmental organisation.

**Table 2:** Weight status according to body mass index (BMI) for children with ASD (*n* = 135).

Weight Status According to BMI	*n* (%)
Underweight (BMI < 5th percentiles)	15 (11.1)
Normal (BMI ≥ 5th to <85th percentiles)	68 (50.4)
Overweight (BMI ≥ 85th to <95th percentiles)	25 (18.5)
Obese (BMI ≥ 95th percentiles)	27 (20.0)

BMI—body mass index.

**Table 3:** Frequency of atypical mealtime behaviours and oral sensory processing difficulties among children with ASD (*n* = 150).

Measures	Classification	*n* (%)	Mean ± SD
Brief Autism Mealtime Behaviour Inventory (BAMBI)—total score			44.6 ± 8.02
	Atypical	144 (96.0)	
	Typical	6 (4.0)	
Oral Sensory Processing (OSP)—total score			25.8 ± 8.18
	Just like the majority of others	70 (46.7)	
	More than others	51 (34.0)	
	Much more than others	29 (19.3)	

SD—standard deviation.

**Table 4:** The correlation between oral sensory processing and mealtime behaviours among children with ASD (*n* = 150).

Relationship	Pearson Correlation (*r*)	*p*-Value
OSP total score and BAMBI total score	0.65	<0.001
OSP total score and sub-domain BAMBI: limited variety	0.64	<0.001
OSP total score and sub-domain BAMBI: food refusal	0.45 ^a^	<0.001

^a^ Spearman correlation.

**Table 5:** Simple linear regression between oral sensory processing, age, severity of ASD symptoms and health-related comorbidities.

Risk Factors	Unstandardised		Standardised	*t*	95% CI	*p*-Value
	B	Std. error	*Beta*			
Oral sensory processing total score	0.643	0.059	0.666	10.814	0.525, 0.760	<0.001 *
Age	−1.111	0.332	−0.266	−3.350	−1.766, −0.455	0.001 *
Severity of ASD symptoms (moderate and severe)	2.963	1.289	0.186	2.299	0.416, 5.511	0.023 *
GI symptoms (e.g., constipation, diarrhoea, abdominal pain, vomiting)	5.199	1.903	0.220	2.732	1.438, 8.960	0.007 *
ADHD	0.010	1.446	0.001	0.007	−2.849, 2.868	0.995

* Significant *p*-value < 0.05. ASD—autism spectrum disorder; GI—gastrointestinal; ADHD—attention deficit hyperactive disorder; CI—confidence interval.

**Table 6:** Results of the multiple linear regression analysis determining the risk factors associated with atypical mealtime behaviours.

Risk Factors	Unstandardised		Standardised	*t*	95% CI	*p*-Value
	B	Std. error	*Beta*			
Oral sensory processing total score	0.608	0.058	0.630	10.483	0.493, 0.722	<0.001 *
Age	−0.694	0.250	−0.166	−2.771	−1.189, 0.199	0.006 *
Severity of ASD symptoms (moderate and severe)	2.585	0.942	0.162	2.743	0.722, 4.448	0.007 *
GI symptoms (e.g., constipation, diarrhoea, abdominal pain, vomiting)	1.543	1.436	0.065	1.074	−1.296, 4.381	0.285

*F* = 47.118, *p* < 0.001; *R^2^* = 0.494 (adjusted *R^2^* = 0.483); stepwise variable method was used; * significant *p*-value < 0.05. ASD—autism spectrum disorder; GI—gastrointestinal; CI—confidence interval.

## Data Availability

The dataset analysed in this study is available on request through the correspondence author.
